# Authors' reply to “Sex and weighting in abbreviated DASI-based perioperative risk prediction”

**DOI:** 10.1016/j.eclinm.2026.104254

**Published:** 2026-10-06

**Authors:** Duminda N. Wijeysundera, Keying Xu, Daniel I. McIsaac, FIT After Surgery Investigators and METS Investigators, S. Abdellatif, M. Abolfathi, G.L. Ackland, T. Ahmad, E. Al Azazi, M. Ali, S.M.H. Alibhai, S. Allen, A. Ambosta, M. Alsaif, N. Ami, L. Andrews, S. Au, S. Avramescu, G. Back, H. Bagry, T. Barnes, S. Bates, W.S. Beattie, N. Beauchamp, C. Beilstein, R. Belliard, E. Black, A. Bodger, P. Bodger, M.F. Boko, C. Bolger, F. Bonazza, B. Borg, M. Bosch, D. Bramley, J. Brannan, R.H. Breau, A. Brown, R. Brull, K. Brunello, D. Campbell, A.M. Carrera, M. Celinski, V. Chan, S. Charummoottil, I. Chergui, T.R. Chesney, S. Choi, H.A. Clarke, A. Collingwood, H. Collins, C. Corriea, T. Creary, C. Crescini, B.L. Croal, J. Dale-Gandar, P. Dalley, M. Davis, L. Day, A. Dent, D. Dillane, J. Dimech, J. Dion, K. Dobson, J. Douglas, S. Drozdz, L.M. Drudi, D. Dumerton, M. Edwards, N.Y. Edwards, S. Ehtesham, H. El Beheiry, A. Elliott, M. Ellis, Y. Essaji, K. Everingham, C. Farrington, A. Ferenc, H. Filipe, A. Fleet, K. Flores, S. Gabriel, S. Gagne, L. Gallego-Paredes, S. Gandotra, M. Gertsman, G. Godsall, A. Goel, K. Golder, J. Grant, J.T. Granton, K. Greaves, J. Green, M.P.W. Grocott, U. Gurunathan, A. Gutierrez Del Arroyo, K. Hagen, A. Hall, C. Hanley, R. Haslop, K. Higgie, G. Hillis, E. Hladkowicz, H. Houston, Y. Hu, A. Hunt, S. Hurford, J. Hutton, H. Ismail, S. Jack, E. Jacobsohn, S. Jain, S. James, R. Jang, M. Januszewska, A. Jason-Smith, S. Jhanji, M. Karizhenskaia, K. Karkouti, L. Kaustov, K. Kenchington, A. Kennedy, E. Kennedy, C. Keown-Stoneman, T. Kerelska, R.K. Kerridge, S. Khandadashpoor, C.J. Kim, C. King, Y. Kirabiyik, M. Koutra, K. Kuang, J. Kumar, J. Kunasingam, S. Kynaston, K.S. Ladha, M. Lalu, H. Lawrence, G. Lebovic, L. Lee, S. Lee, K. Leslie, D. Levett, R. Lifford, H. Lindsay, G. Lorello, M. Lorimer, L. Loughney, M. Louridas, M. Lum, V. Lyon, A. Maccormick, D. Macdonald, N. Macdonald, S.Y. Macdonell, S. Macklin, J. Malherbe, A. Malik, M. Mamdani, F. Marshall, D. Martin, P. Masel, G. Mattina, C.D. Mazer, D. Mcallister, C.J. McCartney, S.A. McCluskey, N. McMillan, J. Mcneil, M. Melo, A. Melville, R. Miller, J.F. Morales, A. Murmane, L. Navarra, E. Niebrzegowska, S. Nnorom, P. Oh, S. Olliff, T.W. Painter, M.L. Pakats, M. Parotto, J. Pazmino-Canizares, S.M. Pereira, M. Phull, M. Pinto, K. Pirie, S. Pitt, H. Poonawala, J. Pretto, M. Puts, A. Raj, N. Rajapakse, R. Raobaikady, A. Reyes, C. Richard, M. Rooms, L. Ruo, S.L. Russell, F. Saad, K. Salmon, J. Samuel, R. Sara, L. Seaward, P.E. Serrano, S. Shaheen, T. Short, M.A. Shulman, N. Siddiqui, H. Sivakumar, P. Sivalingam, B. Smethurst, E. Smith, P. Somascanthan, A.M. Southcott, R. Spence, M. Stanbrook, K. Steele, R.C.M. Stephens, C. Stonell, D. Sussman, N.F. Syeda, J. Tai, N. Tantony, H. Taylor, N.C. Terblanche, M. Tessier, B. Thompson, H. Thompson, M. Thorleifson, K.E. Thorpe, A. Tippett, R. Tod, E. Torres, M. Towns, O. Tronstad, B. Tyrell, J. Van Der Westhuizen, J. Van Vlymen, F. Vandenbroucke-Menu, E. Vowotor, S. Wallace, M. Wasowicz, E. Waymouth, A.C. Wei, J. Whalley, H.C. Wijeysundera, C. Wilde, C. Wong, E. Wright, J. Wu, K. Xu, A. Yagnik, S. Yagnik, M. Yang, A. Zahavich, K. Zarnke

**Affiliations:** a Li Ka Shing Knowledge Institute, Unity Health Toronto, Toronto, Ontario, Canada; b Department of Anesthesiology and Pain Medicine, Temerty Faculty of Medicine, University of Toronto, Toronto, Ontario, Canada; c Strategic Partnerships in Health Excellence, Research, and Engagement, Unity Health Toronto, Toronto, Ontario, Canada; d Departments of Anesthesiology and Pain Medicine, University of Ottawa and The Ottawa Hospital, Ottawa, Ontario, Canada

We thank Ho and colleagues for their thoughtful comments^1^ on our analysis.^2^ We welcome the opportunity to address their questions regarding sex-related differences in the prognostic performance of the Duke Activity Status Index (DASI), the impact of item weighting on the performance of the modified four-item DASI (M-DASI-4Q),^3^ and the potential prognostic value of biological age.

We first evaluated whether biological sex modified the prognostic association between DASI and postoperative outcomes, using both sex × DASI interaction terms and sex-stratified analyses. Although both DASI and female sex were associated with postoperative complications in our original prognostic model (Model 3), there was no statistical evidence of an interaction between sex and DASI (p = 0·84). Consistent with this finding, in analyses stratified by biological sex, the adjusted association between DASI and major complications was essentially identical in males (adjusted odds ratio [aOR] per 5-unit decrease in DASI, 1·07; 95% CI, 1·03–1·11) and females (aOR 1·07; 1·02–1·13).

We similarly found no evidence that biological sex modified the association between DASI and 30-day major cardiac complications or death. The sex × DASI interaction was not statistically significant in either the model (Model 1) incorporating age and the Revised Cardiac Risk Index (p = 0·30) or the model (Model 2) additionally incorporating preoperative natriuretic peptide concentration (p = 0·36). Thus, although previous analyses of the METS cohort identified sex-related differences in the association between objectively measured cardiopulmonary exercise testing performance and postoperative outcomes,^4^ we did not identify corresponding differences when functional capacity was instead assessed using a standardised self-reported instrument such as the DASI.

We next evaluated whether the prognostic performance of the M-DASI-4Q improved when its four component activities retained their original DASI item weights, resulting in a weighted score ranging from 0 to 25·5. The weighted and unweighted M-DASI-4Q scores were almost perfectly correlated (Spearman correlation 0·99). Consistent with this close correspondence, use of the original item weights did not materially improve prognostic performance (Table 1). Across the models, measures including the c-index, fraction of new predictive information, and net reclassification improvement were similar or, if anything, slightly less favourable with the weighted M-DASI-4Q. For example, when added to age and RCRI for prediction of the primary outcome, the fraction of new predictive information was 15·6% with the unweighted M-DASI-4Q versus 13·1% with the weighted version, while the corresponding net reclassification improvement was 0·26 versus 0·23. These findings suggest that the reduced incremental prognostic performance of the M-DASI-4Q compared with the full 12-item DASI is more likely attributable to the reduction in the number of questionnaire items than to equal weighting of the four retained items.

**Table 1:** Additional predictive performance of the unweighted and weighted M-DASI-4Q with respect to primary and secondary outcomes.

Covariates in model	c-index	Likelihood ratio test	Fraction of new predictive information	Net reclassification improvement index (95% CI)
**Primary outcome: 30-day major cardiac complication or death**				
Model 1				
Age, RCRI	0·68 (0·63–0·73)			
+unweighted M-DASI-4Q	0·69 (0·65–0·74)	p = 0·04	15·6%	0·26 (0·08–0·44)
+weighted M-DASI-4Q	0·69 (0·64–0·73)	p = 0·008	13·1%	0·23 (0·08–0·44)
Model 2				
Age, RCRI, abnormal NP^a^	0·70 (0·65–0·74)			
+unweighted M-DASI-4Q	N.A.^b^	p = 0·13	N.A.^b^	N.A.^b^
+weighted M-DASI-4Q	N.A.^b^	p = 0·051	N.A.^b^	N.A.^b^
**Secondary outcome: all-cause major complication**				
Model 3				
Age, sex, surgery type	0·67 (0·65–0·69)			
+unweighted M-DASI-4Q	0·68 (0·66–0·70)	p = 0·001	6·6%	0·14 (0·05–0·22)
+weighted M-DASI-4Q	0·68 (0·66–0·70)	p < 0·0001	5·6%	0·13 (0·04–0·21)

Abbreviations: M-DASI-4Q, four-question (modified) Duke Activity Status Index; N.A., not applicable; NP, natriuretic peptide.

a Defined as B-type natriuretic peptide (BNP) ≥92 ng/L or N-terminal pro-B-type natriuretic peptide (NT-proBNP) ≥200 ng/L.

b Additional measures of incremental prognostic performance were calculated if the likelihood ratio test was statistically significant (p < 0·05).

Finally, we agree with Ho and colleagues that biological age may plausibly better capture some aspects of perioperative risk than chronological age alone. We did not have access to the additional biomarkers required to calculate biological age using approaches such as PhenoAge and therefore could not directly evaluate this hypothesis.^5^ However, DASI may already capture some of the physiological vulnerability associated with biological ageing. In a recent analysis of the FIT After Surgery cohort, DASI scores were strongly correlated with frailty measured using the Clinical Frailty Scale (Pearson R −0·64).^6^ Thus, structured assessment of functional capacity using the DASI may capture overlapping domains of frailty and physiological reserve that are not represented by chronological age alone. Nevertheless, directly evaluating whether measures of biological age provide incremental prognostic information beyond DASI and established perioperative risk predictors would be an important area for future research.

Overall, our additional analyses provide no evidence that the prognostic association of DASI differs materially by biological sex and demonstrate that retaining the original DASI item weights does not improve the prognostic performance of the abbreviated M-DASI-4Q. These findings support use of a common DASI framework across sexes and reinforce the full 12-item DASI as the preferred instrument when structured self-reported functional capacity assessment is incorporated into perioperative risk assessment.

## Contributors

DNW, KX, and DIM developed the methodology. DNW and KX performed the formal analysis. DNW drafted the original manuscript. All authors critically revised the manuscript, approved the final version for submission, and agree to be accountable for the work. All authors had full access to all study data. DNW and KX verified the underlying data and take responsibility for its integrity and accuracy.

## Declaration of interests

DNW and DIM are members of the Board of Directors of the International Anesthesia Research Society, unrelated to this work. DNW has served on advisory boards for Vertex Pharmaceuticals, unrelated to this work. DIM reports participation on a Data Safety Monitoring Board or Advisory Board for PRICE-2, unrelated to this work. All other authors declare no competing interests.
